# The Auxin-Induced Protein Gene (MsARG4) Regulates Rapid Stem Elongation and Nutritional Quality Enhancement in Alfalfa

**DOI:** 10.3390/plants15132028

**Published:** 2026-06-30

**Authors:** Xiaoya Hao, Sisi Huang, Zhike Liu, Shu-An Jia, Linjiao He, Licong Sun, Shiqing Wang, Aiyguli Tuerdi, Tuerxunayi Maimaitiyiming, Yuanqiu Li, Yisilayi Dawuti, Jiangchun Wan, Guili Jin, Jiangjiao Qi

**Affiliations:** 1 College of Grassland Science, Xinjiang Agricultural University, Urumqi 830052, China; 18835106427@163.com (X.H.); 19375102057@163.com (S.H.); he1097888956@163.com (L.H.); 13609180834@163.com (L.S.); 13308054916@163.com (S.W.); 18599279181@163.com (A.T.); tuexay24@xjau.edu.cn (T.M.); xjaulyq@xjau.edu.cn (Y.L.); israyil228@aliyun.com (Y.D.); wanjiangchun1@163.com (J.W.); jguili@126.com (G.J.); 2 College of Animal Science and Technology, Xinyang Agricultural and Forestry University, Xinyang 464000, China; zkeliu@163.com; 3 Animal Disease Prevention and Control Center of Xinjiang Uygur Autonomous Region, Urumqi 830011, China; jasonjia1023@163.com

**Keywords:** *MsARG4*, function, genetic transformation, quality improvement, stem growth

## Abstract

Alfalfa (*Medicago sativa* L.) exhibits high feeding value in agricultural and livestock production, with its above-ground parts determining yield and quality. Promoting the development of molecular breeding of alfalfa helps to clarify the function of *MsARG4* in regulating its growth. Obtaining *MsARG4*-overexpressing and RNA interference-positive plants via genetic transformation, we compared their phenotypic, nutritional, physicochemical, and cellular structural characteristics with those of wild-type plants. The results showed that the plant height, stem diameter, number of branches, number of lateral branches, internode length, and crude protein content of overexpressing plants were significantly higher than those of wild-type and RNAi plants (*p* < 0.05). Furthermore, we observed that the cell area of OE lines was larger than that of WT and RNAi lines. The stem-to-leaf ratio of overexpressing plants in the branching and budding stages was significantly lower than in wild-type and RNAi plants (*p* < 0.05). Our study suggests that *MsARG4* promotes alfalfa quality improvement and rapid stem growth.

## 1. Introduction

The high yield and superior quality of alfalfa (*Medicago sativa* L.) provide stable, protein-rich forage for animal husbandry, serving as a crucial material foundation for the efficient development of modern livestock farming. Stalk elongation is the key factor in establishing the plant’s structural framework, determining biomass yield, and influencing forage quality [1]. Stalks consist of internodes, the length of which can be measured; their elongation and thickening provide spatial support for leaf attachment and photosynthesis, while directly constituting the main dry matter of the above-ground portion of alfalfa during harvesting [2]. Studies have demonstrated that rapid stalk elongation significantly increases plant height and branching levels, optimizes plant architecture, and enhances both harvesting frequency and fresh forage yield per unit area [3].

Stem elongation is a complicated process mediated by several interacting factors. Internode length formation in alfalfa stems results from the synergistic interaction between cell proliferation in the apical meristem and elongation growth of internode cells, involving intricate genetic backgrounds and endogenous hormone interactions [4]. Li et al. demonstrated that stem morphology and lignin content in impatiens are closely correlated with *MYB4* gene expression under different environmental conditions [5]. Xu et al. identified the *PlSPL14* transcription factor in peony as pivotal in regulating cell elongation and division, highlighting that stem elongation influences plant cellular architecture [6]. While Ye et al. revealed that LRM3 in soybeans promotes lignin synthesis by degrading *MYB6,* thereby enhancing stem strength [7], Muhammad et al. through analyzing wheat stem characteristics, further confirmed that mechanical strength and flexibility are critical traits for lodging resistance [8]. Yang et al. found that the *XTH* gene family in osmanthus regulates cell wall relaxation and expansion in response to temperature changes, thereby influencing flowering period control [9].

As a core plant hormone, auxin primarily regulates cell division, elongation, and differentiation, playing a pivotal role throughout the entire plant life cycle, including organogenesis (the initiation of roots, stems, and leaves) and vascular tissue differentiation [10,11]. Plant internode length is influenced by a synergistic interaction of multiple hormones; *OsCYPq1* promotes interspecific elongation in rice by regulating GA synthesis, highlighting the central role of growth hormones in this process [12]. Relevant studies have demonstrated that plant height is jointly regulated by genetic factors, endogenous hormones, and environmental conditions, with GA- and BR-mediated plant hormone signaling pathways holding a dominant position in plant growth and development [13]. Ainoa et al. found that BR exerts multifaceted regulatory effects on plant growth and development along with stress response via mediating transcriptional expression of genes relevant to cell elongation and division [14].

The AUX/IAA protein family serves as a crucial regulatory factor in the plant auxin signaling pathway, present in all plant species [15]. It is abundantly produced in meristems and functions as a core signaling molecule that drives continuous cell division and differentiation, providing the cellular foundation for plant growth and development. Studies have shown that AUX/IAA genes participate in processes such as main root elongation, lateral root formation, and root hair development by regulating the auxin signaling pathway [16]. Research by Roychoudhry et al. revealed that auxin acts as a central regulator in Arabidopsis root development, precisely controlling the growth and development of root tip meristems, as well as cell division, growth, and differentiation, through biosynthesis, transport, and signaling pathways [17]. Ban et al. achieved rapid, precise, and adjustable responses to growth signals by modulating specific AUX/IAA protein degradation rate in Arabidopsis, thereby regulating downstream gene expression [18]. Lan et al. identified differential expression patterns among eight members of the GH3, ARF, and *AUX/IAA* gene families in castor plants through systematic characterization and expression analysis, revealing distinct expression profiles between tall and dwarf cultivars [19]. Combined with proteomic analyses, these differentially expressed *AUX/IAA* and *ARF* genes regulate downstream auxin responses via protein–protein interactions, influencing stem elongation and plant height [15]. Si et al. found that *AUX/IAA* gene expression levels in Dendrobium undergo dynamic changes during flower opening; protein interaction analyses revealed that specific AUX/IAA proteins directly interact with ARF proteins, thereby regulating downstream gene expression to transmit auxin signals [20]. Marzi et al. demonstrated that auxins play a critical role in mediating plant responses to stress conditions like drought and salinization through precise growth regulation [21]. Further, Dong et al. elucidated that the early auxin-responsive genes *SAUR*, *GH3*, and *AUX/IAA* form a multi-layered, precisely feedback-regulated signaling network that is extensively involved in plant development [16].

Although it is known that growth hormones participate in regulating plant height, existing studies have primarily focused on individual traits, with few investigations examining how a single gene collaboratively regulates the dual functions of “rapid stem elongation” and “forage quality”. In particular, the auxin-induced protein gene *MsARG4*, as an AUX/IAA protein family member, has not been systematically characterized in model plants or alfalfa. Therefore, identifying and leveraging key genes like *MsARG4* that synergistically promote alfalfa stem elongation and nutrient accumulation represents a critical approach to balancing the trade-off between alfalfa yield and quality, thereby achieving simultaneous improvements in annual biomass and forage value.

This study employs genetic transformation technologies to systematically elucidate the biological functions of *MsARG4* in alfalfa stem elongation and quality improvement, advancing our understanding of how *MsARG4* mediates rapid growth and enhanced nutritional quality in alfalfa, and providing critical genetic resources and a theoretical basis for developing new alfalfa varieties that balance high yield with superior quality. This research holds significant importance for promoting the sustainable supply of high-quality forage for modern animal husbandry.

## 2. Results

### 2.1. Identification of AUX/IAA Protein Family Members and Construction of an Evolutionary Atlas

**Figure 1:**
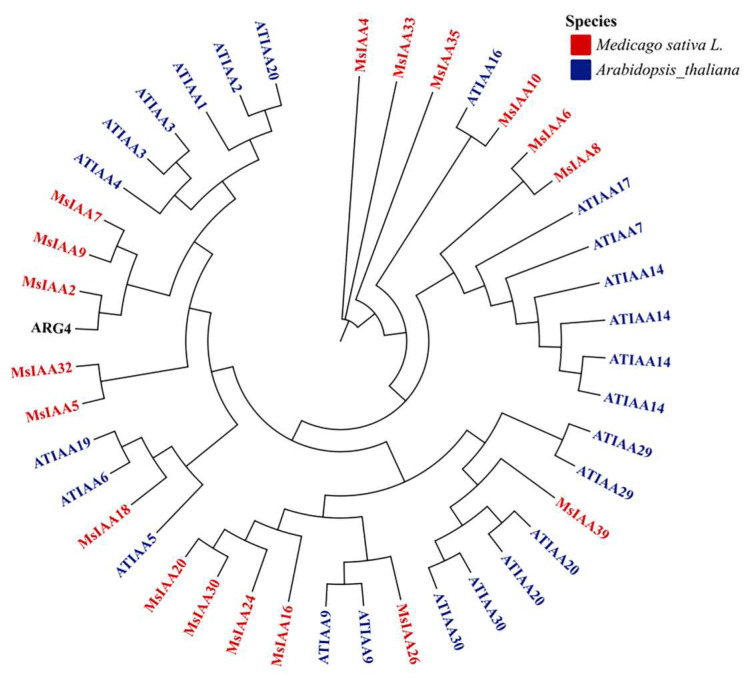
Identification and evolutionary analysis of AUX/IAA family members based on sequence homology. The phylogenetic tree illustrates the phylogenetic relationships of homologous genes between *Medicago sativa* L. (marked in red) and *Arabidopsis thaliana* (marked in blue). Each gene name corresponds to homologous sequences from the two species, where branch lengths and tree topology reflect the genetic distances and evolutionary affinities among these genes.

The phylogenetic tree illustrates the evolutionary relationships among different species (*Medicago sativa* L. and *Arabidopsis thaliana*) (Figure 1; Table S3), with branches leading to ARG4 exhibiting similar lengths to those leading to MsIAA2, MsIAA9, MsIAA7, MsIAA32, MsIAA5, AtIAA19, AtIAA6, AtIAA3, AtIAA2, and AtIAA20, with highly similar homologous protein sequences. In contrast, the branches leading to MsIAA18, AtIAA1, AtIAA4, AtIAA5, MsIAA16, MsIAA26, and MsIAA39 are longer than those leading to ARG4, reflecting greater divergence in their homologous protein sequences. Additionally, the branches leading to MsIAA4, MsIAA33, and MsIAA35 are markedly longer than those leading to ARG4 (Figure 1). MsIAA2, MsIAA9, and MsIAA7 regulate root growth and root system morphogenesis, while MsIAA9 and MsIAA7 also regulate stem and leaf growth.

### 2.2. Analysis of Transcriptional Expression Levels in Alfalfa RNAi Plants, WT Plants, and OE Plants

We selected RNAi plants (RNAi-1, RNAi-5, and RNAi-7 lines), WT plants, and OE plants (OE-1, OE-2, and OE-4 lines) as subjects for our experimental study.

**Figure 2:**
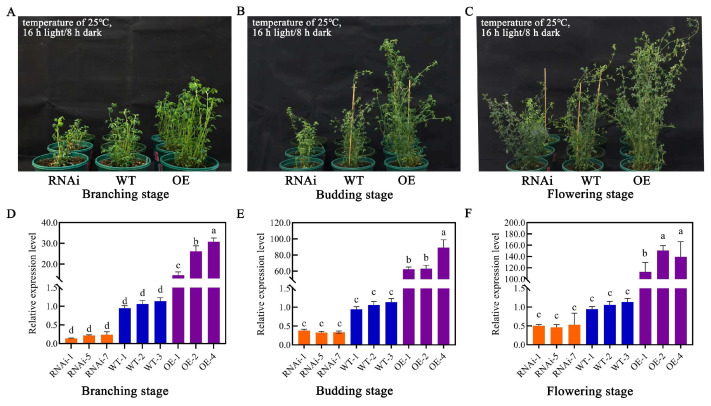
Phenotypic traits and *MsARG4* transcript levels in alfalfa RNAi, WT, and OE plants across developmental stages. This figure illustrates the epigenetic traits and *MsARG4* transcriptional expression levels of different alfalfa lines at various growth stages. (**A**–**C**) show the phenotypic characteristics of RNAi-treated, WT, and OE plants at the branching, budding, and flowering stages, respectively. (**D**–**F**) display the *MsARG4* expression levels of these same plant types at the aforementioned stages. The expression levels of all genes are plotted relative to the expression level of the internal standard (*β-Actin 2*). Different lowercase letters indicate significant differences among lines at each stage (*p* < 0.05), while identical or absent letters signify no significant differences (*p* > 0.05).

Figure 2 shows that the plant height of all alfalfa lines was significantly higher in OE plants than WT and RNAi plants during the branching, budding, and flowering stages (Figure 2A–C), while the overall *MsARG4* transcript levels in OE plants were markedly higher than in WT and RNAi plants (Figure 2D–F; Table S4).

Through the branching, budding and the flowering stages, RNAi, WT, and OE plants exhibited progressively vigorous growth, with the latter demonstrating the most pronounced growth phenotype at the flowering stage (Figure 2A–C). Correspondingly, the *MsARG4* transcript level was highest at the flowering stage, exceeding those observed at both the budding and branching stages (*p* < 0.05) (Figure 2D–F; Table S4). Notably, the *MsARG4* transcript level remained stable across all growth stages in both RNAi and WT plants, and did not differ significantly (*p* > 0.05) (Table S4).

### 2.3. Cell Structure Analysis

**Figure 3:**
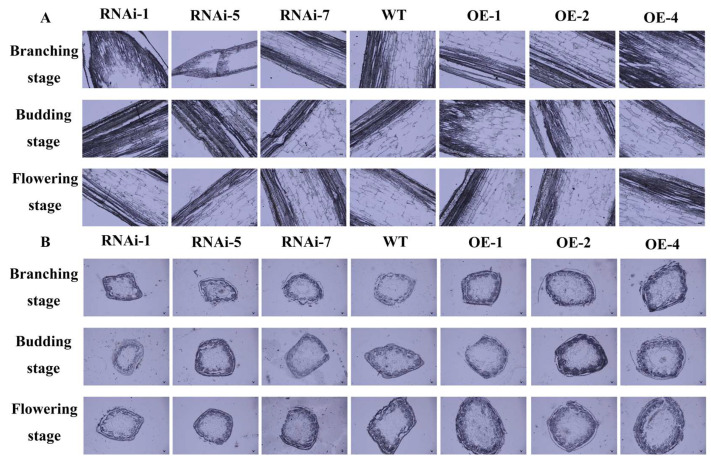
Cell structures of stem tissues in RNAi, WT, and OE plants at different developmental stages. The figures show the cell structure of stem tissues of different alfalfa lines at different growth stages. (**A**) shows longitudinal sections of stem tissues from different plants at various growth stages; (**B**) displays cross-sectional views of stem tissues from the different plants at different developmental stages. Scale: 50 μm.

We selected the low-expression RNAi-1, RNAi-5, and RNAi-7 lines, the wild-type line, and the high-expression OE-1, OE-2, and OE-4 lines as study subjects, with a total of 21 samples collected at the branching, budding, and flowering stages for cellular structure observation (cross-sections and longitudinal sections) (Figure 3A,B).

At the same growth stage, the area and perimeter of RNAi lines and WT cells were significantly smaller than those of OE lines (*p* < 0.05). During the branching and flowering stages, both RNAi and WT cells exhibited significantly smaller diameters than those of OE lines (*p* < 0.05). At the budding stage, the diameter of the cells in the OE-1 and OE-2 lines was significantly larger than that of the OE line (*p* < 0.05), whereas no significant difference was observed between the OE-4 and either WT or RNAi-5 lines (*p* > 0.05). The cell area of OE-1 at flowering was significantly larger than that at branching and budding stages (*p* < 0.05) (Figure 3B and Figure S1B; Table S11(1)). No significant differences in cell area were found among the WT, RNAi-1, and RNAi-7 lines across different growth stages (*p* > 0.05). However, the cell area of RNAi-1 and RNAi-7 increased with advancing growth stage. At the flowering stage, the cell area, perimeter, and diameter of the OE-1 and OE-4 lines were significantly larger than those at the budding stage (*p* < 0.05) (Figure 3B and Figure S1B; Table S11(1)).

At the branching stage, no significant differences in cell area were observed among RNAi lines, wild-type, and OE lines (*p* > 0.05); however, the cell area of the OE lines was larger than that of both the RNAi lines and the WT line. At the budding stage, the cell area of the OE-2 and OE-4 lines was significantly larger than that of the RNAi lines (*p* < 0.05). At the flowering stage, the cell area of the OE lines was significantly larger than that of the RNAi lines (*p* < 0.05). Except for RNAi-7, no significant differences in cell perimeter were detected between the OE and RNAi lines across all growth stages (*p* > 0.05). No significant differences were observed in diameter between the OE and RNAi lines at either the budding or flowering stages (*p* > 0.05) (Figure 3A and Figure S1A; Table S11(2)). The cell area of the OE lines at the flowering stage was significantly larger than that at the branching stage (*p* < 0.05). Across different growth stages, no significant differences in cell area were found between the RNAi-1 and RNAi-7 lines (*p* > 0.05), but the cell area increased with growth stages. The cell perimeter of the OE-1 lines at both the flowering and budding stages was significantly larger than that at the branching stage (*p* < 0.05) (Figure 3A and Figure S1A; Table S11(2)).

### 2.4. Phenotypic Trait Characterization of Alfalfa RNAi, WT, and OE Plants Across Growth Stages

**Figure 4:**
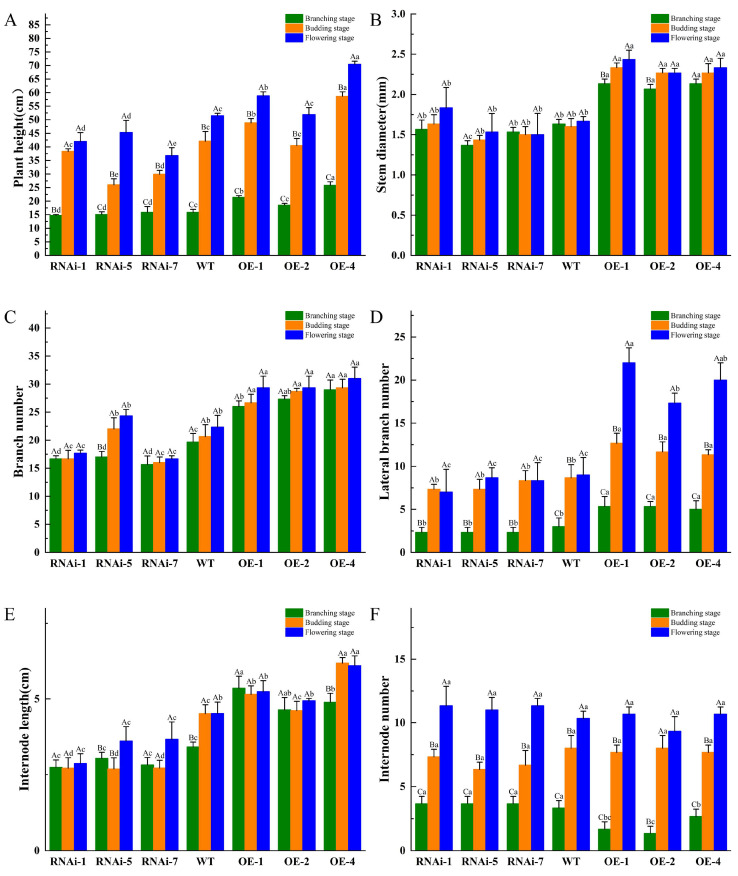
Phenotypic character analysis of different lines of alfalfa. The image displays the phenotypic traits of alfalfa plants from different lines at various growth stages. The traits measured include plant height (**A**), stem diameter (**B**), number of branches (**C**), lateral branches (**D**), internode length (**E**), and number of internodes (**F**) during the branching, budding and flowering stages. Different lowercase letters indicate significant differences among lines at the same stage (*p* < 0.05); different uppercase letters indicate significant differences within a line at different stages (*p* < 0.05); identical or absent letters denote no significant difference (*p* > 0.05).

Three plants were randomly selected from each line, totaling 21 samples for phenotypic trait determination. At all growth stages, OE plants showed significantly greater plant height, stem diameter, branch number, lateral branch number, and internode length than WT and RNAi plants (*p* < 0.05) (Figure 4A–E; Table S5). During the branching stage, OE plants had a significantly lower number of internodes than WT and RNAi plants (*p* < 0.05) (Figure 4F; Table S5). At different growth stages, OE-2 line plant height was dramatically higher than RNAi-7 line plants (*p* < 0.05), while showing no apparent disparity from the WT line (*p* > 0.05) (Figure 4A). At various growth stages, WT plant stem diameter did not differ significantly from the RNAi-1 line (*p* > 0.05), but differed dramatically from all other lines (*p* < 0.05) (Figure 4B). At the budding stage, the branch number in WT plants showed no obvious disparity from that of the RNAi-5 line (*p* > 0.05) (Figure 4C), while at different growth stages, lateral branch number in WT plants showed no marked disparity from those of RNAi plants (*p* > 0.05) (Figure 4D). At the budding and flowering stages, the number of internodes in OE-2 plants showed no marked disparity from that of WT (*p* > 0.05) (Figure 4E), while that of OE plants did not differ significantly from both WT and RNAi plants (*p* > 0.05) (Figure 4F).

At the flowering stage, WT, RNAi, and OE plants showed significantly greater plant height and internode number than at the budding and branching stages *(p* < 0.05) (Figure 4A,F; Table S5). In WT and OE plants, lateral branch number at the flowering stage was significantly greater than at the branching and budding stages (*p* < 0.05); RNAi plants did not differ significantly in lateral branch number between the flowering and budding stages (*p* > 0.05) (Figure 4D; Table S5). Stem diameter did not differ significantly between WT and RNAi plants across different growth stages (*p* > 0.05) (Figure 4B; Table S5). At the flowering stage, RNAi-5 branch number and internode length were significantly higher than at the branching and budding stages (*p* < 0.05) (Figure 4C,E; Table S5). No significant difference in branch number was observed among OE plants across different growth stages (*p* > 0.05) (Figure 4C),while the stem diameter in the OE-1 and OE-2 lines at the flowering and budding stages was significantly higher than at the branching stage (*p* < 0.05) (Figure 4D). Additionally, OE-4 internode length was significantly higher at the flowering and budding stages than at the branching stage (*p* < 0.05) (Figure 4E; Table S5).

### 2.5. Nutritional Quality Analysis of Alfalfa RNAi, WT, and OE Plants

Three plants were randomly selected from each line, and 21 above-ground parts were collected at the flowering stage for nutritional parameter determination.

While crude protein (CP) and relative feed value (RFV) contents in OE plants were significantly higher than in WT and RNAi plants (*p* < 0.05), no significant difference was found between the latter two (*p* > 0.05) (Table 1 and Table S6). Stem-to-leaf ratio (SLR) in RNAi plants was significantly higher than that in WT and OE plants (*p* < 0.05), and the neutral detergent fiber (NDF), crude ash (Ash), and dry matter (DM) contents of RNAi plants were significantly higher than those in OE plants (*p* < 0.05) (Table 1 and Table S6). Regarding NDF content, RNAi plants showed significantly higher values than in OE plants, but did not differ significantly compared with WT plants (*p* > 0.05). In acid detergent fiber (ADF) content, RNAi plants showed significantly higher values than in OE plants, whereas WT plants showed significantly higher values than RNAi plants (*p* < 0.05) (Table 1 and Table S6), and no significant difference was observed in ether extract (EE) content among OE, WT, and RNAi plants (*p* > 0.05) (Table 1 and Table S6). Additionally, no significant differences were observed between CP, EE, NDF, ADF, Ash, and SLR among different lines of the same genotype (*p* > 0.05) (Table 1 and Table S6). The DM content of the OE-4 line was significantly higher than in the OE-2 line (*p* < 0.05), and that of the RNAi-1 was significantly higher than in the RNAi-5 and RNAi-7 lines (*p* < 0.05) (Table 1 and Table S6).

### 2.6. Analysis of Hormone Differences Among Different Lines of Alfalfa

With three plants randomly selected from each line, 63 stem tissue samples and 63 leaf tissue samples (21 per stage for each stage) were collected at the branching, budding, and flowering stages, respectively, totaling 126 stems and leaf samples used for IAA, CTK, GA, and BR assays.

**Figure 5:**
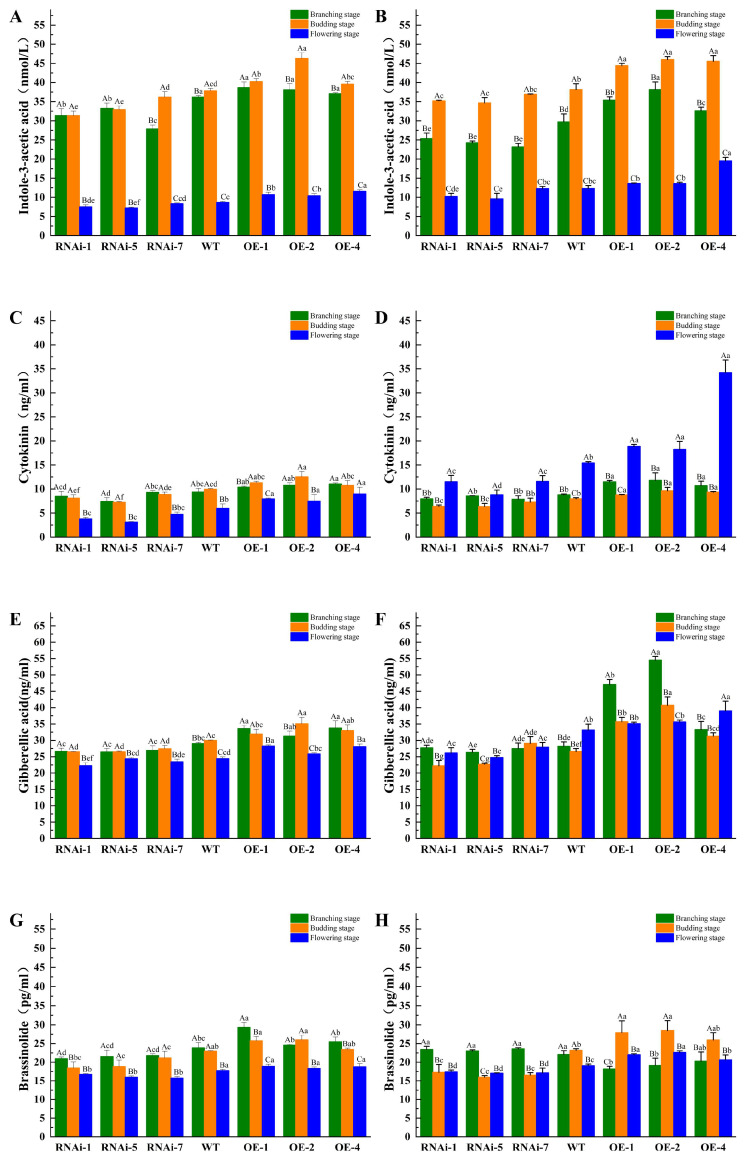
Differential analysis of growth hormones in alfalfa. Analysis of growth hormone differences among different alfalfa lines at various growth stages. (**A**,**C**,**E**,**G**) represent the IAA, CTK, GA, and BR contents in stem tissues, respectively; (**B**,**D**,**F**,**H**) represent the IAA, CTK, GA, and BR contents in leaf tissues, respectively. Different lowercase letters indicate significant differences among lines at the same stage (*p* < 0.05), different uppercase letters indicate significant differences within the same line at different stages (*p* < 0.05), and identical letters denote no significant differences (*p* > 0.05).

During the branching and budding stages, IAA and GA levels in OE stem tissues were significantly higher than in RNAi plants (*p* < 0.05). At the branching stage, CTK levels in the OE-4 line were significantly higher than that in RNAi plants (*p* < 0.05), while those in OE-1 and OE-2 were significantly higher than in the RNAi-1 and RNAi-5 lines (*p* < 0.05). BR levels in the OE-1 and OE-4 lines were significantly higher than in RNAi plants (*p* < 0.05), whereas no significant difference was found between OE-2 line and RNAi plants (*p* > 0.05) (Figure 5; Table S7).

During the budding and flowering stages, CTK levels in OE plants were significantly higher than in RNAi (*p* < 0.05). BR levels in the OE-1 and OE-2 lines were significantly higher than in RNAi plants (*p* < 0.05), whereas the OE-4 line and WT lines did not differ significantly (*p* > 0.05) (Figure 5; Table S7).

At the flowering stage, IAA levels in OE plants were significantly higher than in RNAi plants (*p* < 0.05). GA levels in the OE-1 and OE-4 lines were significantly higher than in WT plants and RNAi plants (*p* < 0.05), whereas we did not observe a significant difference between the OE-2 line and WT plants or RNAi-5 lines (*p* > 0.05). While BR levels in the OE lines were significantly higher than in RNAi plants (*p* < 0.05), no significant difference was found between the OE lines and WT lines (*p* > 0.05) (Figure 5; Table S7).

During branching and budding, the leaf contents of IAA, CTK, and GA in OE plants were significantly higher than in WT and RNAi plants (*p* < 0.05) (Figure 5; Table S7). In contrast, the leaf BR content in WT and RNAi plants was significantly higher than that in OE plants (*p* < 0.05) (Figure 5H; Table S7). At the budding stage, there was no significant difference in BR content observed between OE and WT plants (*p* > 0.05), but both were significantly higher than RNAi plants (*p* < 0.05) (Figure 5H; Table S7). At the flowering stage, IAA content in OE and WT plants was significantly higher than that in the RNAi-1 and RNAi-5 lines, while this did not differ significantly among the OE-1, OE-2, WT, and RNAi-7 lines (*p* > 0.05) (Figure 5B; Table S7). CTK and BR leaves in OE plant were significantly higher than in WT and RNAi plants (*p* < 0.05) (Figure 5D,H; Table S7), and GA leaves in OE and WT plant did not differ significantly (*p* > 0.05), though both were significantly higher than that in RNAi plants (*p* < 0.05) (Figure 5F; Table S7).

At the flowering stage, stem contents of IAA, CTK, GA, and BR, as well as leaf IAA content, were significantly lower in OE, WT, and RNAi plants than at the branching and budding stages (Figure 5A–C,E,G; Table S7). Leaf CTK content in OE, WT, and RNAi plants at the flowering stage was significantly higher than at the budding stage (*p* < 0.05) (Figure 5D; Table S7), while leaf BR content in OE and WT plants at the budding stage was significantly higher at the budding stage than at the branching and flowering stages *(p* < 0.05); meanwhile, leaf BR content in RNAi plants at the branching stage was significantly higher than at the budding and flowering stages (*p* < 0.05) (Figure 5H; Table S7).

Interestingly, at the branching stage, IAA and CTK levels in stems and leaves were similar among OE, WT, and RNAi plants. During budding stage, IAA and CTK levels exhibited an increasing trend, whereas at the flowering stage, stem tissue showed lower IAA and CTK levels than leaf tissue (Figure 5; Table S7). At the same growth stage, RNAi and WT plants exhibited comparable BR levels in stems and leaves; however, OE plants demonstrated higher BR levels in stems than leaves at the branching stage, while BR levels in both stems and leaves were lower during the budding and flowering stages (Figure 5H; Table S7).

### 2.7. Analysis of Differences in SP and SS Content Among Different Lines of Alfalfa

With three plants randomly selected from each line, 63 stem and 63 leaf tissue samples (21 per stage for each stage) were collected at the branching, budding, and flowering stages, respectively. A total of 126 tissue samples from stems and leaves were used for SS and SP detection.

**Figure 6:**
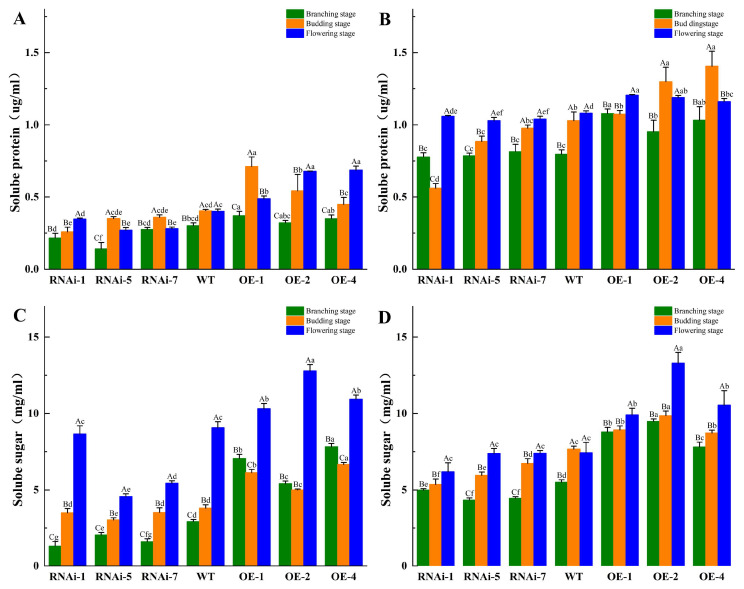
Analysis of differences in SP and SS contents between lines of alfalfa. Analysis of differences in soluble substances among various alfalfa lines across different growth stages. (**A**,**C**) show the SP and SS contents in stem tissues, respectively; (**B**,**D**) show the SP and SS contents in leaf tissues, respectively. Different lowercase letters indicate significant differences among lines at the same growth stage (*p* < 0.05); different uppercase letters indicate significant differences within the same line across different growth stages (*p* < 0.05); identical letters denote no significant differences (*p* > 0.05).

At the branching stage, OE plants had significantly higher stem SP and SS contents than RNAi plants (*p* < 0.05), and the OE-1 line also showed significantly higher stem SP content than WT plants (*p* < 0.05). At the budding stage, both the OE-1 and OE-2 lines showed significantly higher stem SP and SS contents than both WT and RNAi plants (*p* < 0.05); similarly, at the flowering stage, OE plants showed significantly higher stem SP and SS contents than WT and RNAi plants (*p* < 0.05) (Figure 6A,C; Table S8).

At the branching stage, OE plants showed significantly higher leaf SP and SS contents than WT and RNAi plants (*p* < 0.05), while at the budding stage, leaf SS content in OE plants remained remarkably higher than that in WT and RNAi plants (*p* < 0.05); both the OE-1 and OE-2 lines had significantly higher leaf SP contents than WT and RNAi plants (*p* < 0.05). At the flowering stage, leaf SP and SS contents in OE plants were also remarkably higher in WT and RNAi plants (*p* < 0.05), whereas the leaf SS content of WT plants did not differ significantly from that of RNAi plants (*p* > 0.05) (Figure 6B,D; Table S8).

At the flowering stage, the SP content in OE, WT, and RNAi plants (excluding RNAi-7 line) was significantly higher than at the branching stage (*p* < 0.05) (Figure 6A,B), while SS content in RNAi plants at the flowering stage was significantly greater than at the budding and branching stage (*p* < 0.05) (Figure 6C–D). In leaves of OE, WT, and RNAi plants, SS content increased with advancing growth stages in the following order: flowering stage > budding stage > branching stage (Figure 6D; Table S8). Notably, at the same growth stage, SP and SS contents in stems of OE, WT, and RNAi plants were generally lower than in leaf tissues.

## 3. Discussion

This study constructed a phylogenetic tree of gene families encompassing alfalfa and the model plant Arabidopsis thaliana, detailing the specific branching position of *MsARG4* within the tree and providing direct evolutionary evidence for subsequent functional investigations. Zheng et al. demonstrated that *ED1/BnaC05 IAA7* mediates the interaction between BnaARF8 and BnaBZR1, precisely regulating the elongation process of rapeseed stems [22]. Wang et al. demonstrated that downregulation of the IAA9 gene in tomato controls stem elongation and increases plant height [23], while Varaud et al. confirmed that ATIAA2 interacts with ARF8 to inhibit petal growth in Arabidopsis [24]. Additionally, Xi et al. revealed that the ATIAA3-mediated photoreceptor signaling pathway is directly linked to the auxin signaling pathway and is regulated by the light-responsive PIF transcription factor; ATIAA3 deficiency induces elongation phenotypes in the plant hypocotyl under varying light intensities [25]. This study revealed that *MsARG4* shows close phylogenetic relatedness with MsIAA2, MsIAA9, MsIAA7, ATIAA3, and ATIAA2 on the phylogenetic tree, with highly similar protein sequences (Figure 1; Table S4). While MsARG4 belongs to the AUX/IAA family and functions with ARF, GH3 and SAUR to mediate auxin signaling [16], related ARF genes have been systematically studied in maize [15], suggesting that MsARG4 may interact with ARF to regulate downstream GH3 and SAUR expression. Notably, gene transcription does not always correspond to protein accumulation, as observed in AUX/IAA, ARF and GH3 families [19]. Based on these findings, it is inferred that ARG4 may play a similar role in alfalfa growth, and further protein validation is required to fully clarify its biological function.

Plant height is one of the key agronomic traits of alfalfa and a critical indicator of yield improvement [26]. Previous studies have shown that overexpression of *BnaEXPA5*, the rapeseed homolog of IAA7, leads to stem elongation in Arabidopsis, thereby increasing plant height [27]. Auxin stimulates fiber cell development in ovules cultured in vitro, and final fiber length is positively correlated with IAA levels [28]. This study found that plant height in OE plants was remarkably superior to that in WT and RNAi plants at all growth stages (Figure 2 and Figure 4A; Table S5), consistent with previous findings. Furthermore, Qi et al. reported that the cell area of the dwarf elephant grass line CpMott was only 56.4% of that in the tall line CpGMY [29].Our results showed that OE lines exhibited larger cell areas than the RNAi and WT lines (Table S11(1), Figure S1), which is one of the reasons that may account for the significantly greater plant height of OE lines relative to RNAi lines. Stem diameter is closely related to plant mechanical strength and also acts as an effective morphological index for assessing its nutritional status [30]. Sun et al. found that *PlIAA9* exhibits high transcriptional expression levels in the vascular tissue of peony stems, regulating secondary stem growth and thereby increasing stem diameter [10]. Moreover, Xu et al. found that *PtoIAA9m* is highly expressed in the cambial zone and the neighboring young secondary xylem cells of poplar stems, thereby promoting stem thickening [31]. While the number of branches and lateral branches are two key factors determining plant yield [1], Egolan et al. confirmed that the *CmF-308* gene promotes the development of lateral roots in tomatoes; high transcriptional expression of *CmF-308* in the phloem leads to increased branch and lateral shoot numbers as well as delayed flowering [32]. Fan et al. confirmed that phytoplasma infection disrupts auxin signaling in Paulownia, leading to abnormal expression of specific *PfAux/IAA* genes and resulting in excessive branching in white-flowered Paulownia [33]. Internode length is a key trait determining plant height and morphological structure [34]. Researchers in related fields have found that the *PheAUX/IAA34* gene promotes increased internode length in bamboo, thereby regulating plant height [35]. The *PpIAA19* gene increases the number of lateral roots and internode length in tomatoes, regulating plant height [36]. We found that transcriptional expression levels of *MsARG4* in OE plants were remarkably superior overall to those in WT and RNAi plants (Figure 2D–F; Table S4). Such findings conform to past research and explain why the stem diameter, branch number, lateral branch number, and internode length of OE plants exhibited significantly higher values than WT and RNAi plants (Figure 4B–F; Table S5). Related studies have demonstrated that high expression of auxin-responsive genes such as AUX/IAA promotes the development of fibrous roots in *p. heterophylla* and enhances its yield [37], while the *Arabidopsis thaliana* IAA17/AXR3 mutant increases the number of rosette leaves [38]. This study demonstrated that *MsARG4* enhances both alfalfa yield and nutritional quality, while the dual functional mechanism of *MsARG4* requires further elucidation. The potential biological functions of *MsARG4* can enhance alfalfa yield and forage quality, providing novel genetic resources with both theoretical and practical value for high-yield and high-quality breeding.

Plant hormones are key endogenous signaling molecules that regulate diverse developmental stages, participating extensively in essential events including cell division, differentiation, elongation, and organogenesis [39]. IAA is a pivotal hormone regulating cell elongation and plant growth, working synergistically with other plant hormones to modulate plant development [40]. Jin et al. observed that exogenous IAA application remarkably upregulated synthesis of growth-related hormones (e.g., IAA, GA3, CTK, and BR) and signal transduction-related genes in red lilac, thereby promoting seedling growth [41]. GA promotes cell elongation and division, regulating internodal elongation of plant stems and determining plant height [42]. Fang et al. showed that *WRKY114* overexpression in rice reduced GA levels, consequently decreasing plant height [43]. This study revealed that during the branching and bud formation stages, the stem tissues of OE plants exhibited significantly higher IAA and GA concentrations compared to RNAi plants (Figure 5; Table S7), which explains why OE plants showed significantly greater plant height, branching number, and lateral branch count compared to WT and RNAi plants (Figure 5A–E; Table S7). CTK regulates cell division, lateral budding, and leaf senescence, and acts synergistically with IAA [11,40]. Hwang et al. confirmed that CTK and IAA exhibit precise, antagonistic interactions in shoot tips and root embryonic stem cells [44], while we found that the CTK content in the stem tissues of OE plants at the budding and flowering stages was apparently superior to in RNAi plants (Figure 5; Table S7), which explains why OE plants exhibited a significantly superior lateral branch number to WT and RNAi plants (Figure 4A–E; Table S5). Additionally, this study revealed that the IAA and CTK levels in stem and leaf tissues were similar among OE, WT, and RNAi plants during the branching stage; both IAA and CTK levels showed an increasing trend at the budding stage, while at the flowering stage, stem tissue hormone levels were lower than in leaf tissue (Figure 5; Table S7). This suggests that IAA and CTK exert a more pronounced promoting effect on stem growth, thereby conferring superior growth performance to OE plants. The AUX/IAA factor acts as a transcriptional inhibitor in the auxin signaling pathway mediated by ARF proteins [23]. These findings contradict previous studies, and further research is required to elucidate the growth mechanisms mediated by *MsARG4* and its associated auxin-responsive genes.

SS and SP provide the energy and material basis for stem cell growth, synergistically promoting both longitudinal elongation and lateral thickening of the stem [45]. Brian et al. found that a sharp decrease in the expression of the key sucrose synthase gene *SbSUS4*, coupled with significant sucrose accumulation and downregulation of cell wall synthesis-related genes, led to sorghum stem elongation cessation [46]. Wei et al. demonstrated that overexpression of the poplar xyloligosaccharide synthase gene *PsnSuSy2* in tobacco enhanced plant height and stem diameter [47]. Our study revealed that OE plants at the branching and flowering stages exhibited significantly higher stem SP and SS contents compared to RNAi plants (Figure 6; Table S8), consistent with previous findings. Additionally, SS supplies energy to plants and serves as a signaling molecule regulating growth and stress resistance [48], while SP participates in osmotic regulation, enzymatic catalysis, and metabolic processes; their synergistic action maintains cellular homeostasis and promotes plant growth and development [49]. Therefore, the mechanistic role of *MsARG4* in alfalfa stress resistance will become a central focus of our future studies.

## 4. Materials and Methods

### 4.1. Evolutionary Map Construction

Based on bioinformatics analysis of these sequences, a total of 43 candidate sequences were identified by applying the following criteria: amino acid sequence similarity to the query sequence below 85% (all candidate sequences were in the nr database), amino acid sequence length exceeding 200 amino acids (aa), and the presence of a complete open reading frame (ORF). Subsequently, phylogenetic trees were constructed for these 43 candidate sequences using MEGA 7 software.

### 4.2. Total RNA Extraction, cDNA Synthesis, and Primer Design for MsARG4

Total RNA was isolated from alfalfa with the MiniBEST Plant RNA Extraction Kit (Takara, Beijing, China), with its concentration and purity were measured using NanoDrop 2000 (Thermo Fisher Scientific, Wilmington, USA). Using total RNA as the template, cDNA was synthesized according to instructions for the PrimeScript™ IV 1st Strand cDNA Synthesis Kit with the gDNA Eraser (Takara, Beijing, China) (Table S1). Using Primer 5.0 software, we designed three primers (*MsARG4*, *MsARG4*-OE and *MsARG4*-RNAi) for *MsARG4* (Table S1), synthesized by Sangon Biotech (Shanghai, China).

### 4.3. Construction of PEG100-eGFP-OE-MsARG4 and PEG100-RNAi-MsARG4 Vectors

A BLAST alignment was performed between the target sequence and the ‘Zhongmu No.1’ genome (http://alfalfagedb.liu-lab.com/genome_zm01/) (accessed on 24 May 2026). with an E-value threshold of 0.001. The results, presented in Supplementary Table S10, show that the target sequence exhibits high specificity within the genome, suggesting that the RNAi vector constructed based on this sequence possesses strong target specificity and is suitable for further experiments (Table S10).

Using cDNA as the template, *MsARG4* was amplified (Table S1) and analyzed by agarose gel electrophoresis. The OE-*MsARG4* and RNAi-*MsARG4* fragments were recovered using the Gel Extraction Kit (Takara, Beijing, China) and assessed for purity and concentration using NanoDrop 2000. These fragments were inserted into the T vector to construct the OE-T-*MsARG4* and RNAi-T-*MsARG4* plasmids (Table S1). The plasmids were transformed into DH5α chemically competent cells (Takara, Beijing, China), with positive strains confirmed by PCR and sent to the sequencing company (Sangon Biotech, Shanghai, China) for sequencing (Table S3). The *MsARG4* sequence was analyzed using DNAMAN software (v9.0, Lynnon Corporation, San Ramon, CA, USA).

The T-OE-*MsARG4* and T-RNAi-*MsARG4* plasmids were purified using the Plasmid Mini Kit (Takara, Beijing, China), their purity and concentration determined using the NanoDrop 2000. The restriction enzyme recognition sites in the pEG100-OE vector were XbaI and SmaI, while the recognition site in the pEG100-RNAi vector was BsaI. The plasmids T-OE-*MsARG4*, T-RNAi-*MsARG4*, pEG100-OE, and pEG100-RNAi were digested with the respective enzymes (Table S1), and the pEG100-eGFP-OE-*MsARG4* and pEG100-RNAi-*MsARG4* vectors were prepared.

### 4.4. Development of PEG100-eGFP-OE-MsARG4 and PEG100-RNAi-MsARG4 Transgenic Alfalfa Plants

The plasmids pEG100-eGFP-OE-*MsARG4* and pEG100-RNAi-*MsARG4* were introduced into *Agrobacterium tumefaciens* strain GV3101 (Sangon Biotech, Shanghai, China), with the positive transformants confirmed using PCR and sequenced (Table S2). The pEG100-eGFP-OE-*MsARG4* and pEG100-RNAi-*MsARG4* plasmids were transformed into “Zhongmu No.1” alfalfa via an Agrobacterium-mediated method. Transgenic plants were screened to identify positive plants, and high-expression OE and low-expression RNAi plants were identified using primer *(MsARG4*).

### 4.5. Plant Material Selection and Growth Conditions

Healthy wild-type (WT), OE (OE-1, OE-2, and OE-4 lines), and RNAi plants (RNAi-1, RNAi-5, and RNAi-7 lines) at the budding stage were selected and propagated by stem cutting to obtain 8–10 viable plants per line. Cultivation was performed in an illumination climate chamber (25 °C, 60% relative humidity, 16 h light/8 h dark) (model GXZ-500D; Ningbo Jiangnan Instrument Factory, Ningbo, China) with flower pots measuring 16 cm × 16 cm, and the growth medium consisted of nutrient soil and vermiculite at a 3:1 (*v*/*v*) ratio. Cultivation management practices—including watering, pest control, and weed removal—were standardized across all lines.

To ensure rigorous, reasonable, and reproducible data collection, WT, OE, and RNAi plants were uniformly cut for the first time when they grew to the budding stage. For subsequent phenotypic and nutritional analyses, three plants per line were randomly selected at the branching, budding and flowering stages. Tissue samples were collected from the mid-part of the stem (excluding the base and apex regions) and fully expanded leaves, then rapidly frozen in liquid nitrogen and stored in a −80 °C ultra-low-temperature freezer for subsequent physiological, qRT-PCR, and cellular structure analysis. *β-Actin 2* (F:GATGCTGAGGATATTCAACCCC, R:CCATGACACCAGTATGACGAGG) was used as the internal reference gene, while the primers in Table S1 were used for qRT-PCR, which was completed using the LightCycler96/LightCycler480system. The relative expression of each gene was calculated according to Kenneth report [50].

### 4.6. Cell Morphology Observation

Stem segments of uniform length were harvested from the mid-section of the stem of WT, OE, and RNAi plants at different growth stages. To examine cell morphology and stem anatomy, paraffin tissue sections were prepared without staining and observed using a microscope (Nikon Corporation, Tokyo, Japan). Longitudinal sections were observed at 10 × 10 magnification, and transverse sections at 10 × 4 magnification. Representative images were captured at each developmental stage to compare cellular architecture across the three genotypes.

### 4.7. Phenotypic Trait Assessment

We measured plant height from the base to the apex with a tape measure, and stem diameter at the midpoint of the main stem using a vernier caliper (DWKC-2012, Anhui, China). Branch number was determined by counting the total number of primary branches per plant, while lateral branch number was determined by counting the number of effective branches on the primary branches. Internode number was determined according to the number of stem segments on the primary branches, and internode length was determined by measuring the average length between adjacent internodes on the primary branches.

### 4.8. Analysis of Nutritional Traits

Samples of WT, OE, and RNAi plants were dried to constant weight. the percentage of which, relative to fresh weight, was recorded as dry matter (DM) content; the stem-to-leaf dry weight ratio was recorded as the stem-to-leaf ratio (SLR). Contents of crude protein (CP), crude ash (Ash), crude fat (ether extract, EE), acid detergent fiber (ADF), and neutral detergent fiber (NDF) were determined according to GB/T 6432-1994, GB/T 6438-2007, GB/T 14772-2008, NY/T 1459-2007, and GB/T 20806-2007 methods [51,52,53,54,55], respectively, and each specimen was analyzed in triplicate, with the mean value was calculated. The relative feed value was determined using the formula RFV = (120/NDF) × (88.9 − 0.799 × ADF)/1.29.

### 4.9. Physiological and Biochemical Trait Assays

Samples (stems and leaves) were taken from a −80 °C ultra-low-temperature freezer, ground in liquid nitrogen, and then washed three times with PBS buffer (pH 7.3). The samples were fully collected, and the cell concentration was finally adjusted to 1 × 10^6^ cells/mL for the determination of IAA, CTK, GA, BR, and SP contents. For SS content determination, the sample tissues were re-ground, washed three times with 80% ethanol, and fully recovered.

The contents of IAA (ELISA kit JM-01121P1, Jiangsu, China), CTK (ELISA kit JM-01038P1, Jiangsu, China), GA (ELISA kit JM-01074P1, Jiangsu, China), and BR (ELISA kit JM-0156Z1, Jiangsu, China) were determined and are expressed in nmol/L, ng/mL, pmol/L, and pg/mL, respectively. The content of soluble proteins (SP) was measured using the BCA Protein Quantification Kit (SB-WB013; Shanghai, China) and expressed in μg/mL, with determination ranges as follows (and HRP as the internal standard unless otherwise specified): IAA, 1–48 nmol/L; GA, 1–120 pmol/L; CTK, 1–48 ng/mL; BR, 15–240 pg/mL; and SP, 20–2000 μg/mL (standard: BSA).

The content of soluble sugars (SS) was measured using a soluble sugar kit (ADS-W-TDX039; Jiangsu, China) and expressed in mg/mL, with the determination range for SS being 0–280 mg/mL (standard: anhydrous glucose).

### 4.10. Statistical Analyses

Data were analyzed by one-way ANOVA, and Duncan’s multiple range test was used for post hoc comparisons (*p* < 0.05) in SPSS Statistics version 20.0 (SPSS Inc.; Chicago, IL, USA). Nutritional traits data were denoted as the mean ± standard deviation, and graphs were generated using OriginPro 2024 (OriginLab Corp., Northampton, MA, USA).

## 5. Conclusions

This research showed that *MsARG4* significantly increased alfalfa plant height, stem diameter, branch number, and internode length. Moreover, the overexpression of *MsARG4* significantly elevated the levels of CP, SP, and SS, and decreased the NDF and ADF in alfalfa, while *MsARG4* also increased both the area and the perimeter of alfalfa stem cells. Our research shows that *MsARG4* promotes rapid stem development and elevates forage quality in alfalfa. New gene resources are required that provide theoretical value and application potential for later elucidation of breeding for high yield and high quality.

## Figures and Tables

**Table 1:** Nutritional Quality Analysis of The Flowering Stage in RNAi Plants, WT Plants, and OE Plants.

Plant Nutritional Index	CP (%)	EE (%)	NDF (%)	ADF (%)	DM (%)	Ash (%)	SLR (%)	RFV
**OE-1**	23.58 ± 2.16 ^a^	3.14 ± 1.20 ^a^	41.17 ± 0.93 ^b^	28.80 ± 1.52 ^cd^	47.08 ± 0.58 ^de^	12.24 ± 1.02 ^c^	55.30 ± 2.28 ^c^	150.22 ± 2.60 ^a^
**OE-2**	23.20 ± 1.58 ^a^	3.22 ± 1.08 ^a^	40.48 ± 0.62 ^b^	27.75 ± 0.66 ^d^	41.06 ± 1.83 ^e^	11.99 ± 1.48 ^c^	55.75 ± 0.74 ^c^	154.67 ± 3.51 ^a^
**OE-4**	22.53 ± 0.91 ^a^	3.56 ± 0.69 ^a^	40.38 ± 0.71 ^b^	29.06 ± 0.89 ^cd^	54.12 ± 1.55 ^d^	13.81 ± 0.76 ^bc^	57.53 ± 0.90 ^c^	152.72 ± 3.61 ^a^
**WT**	18.65 ± 1.22 ^b^	2.62 ± 0.63 ^a^	42.29 ± 0.94 ^ab^	37.63 ± 0.58 ^a^	58.96 ± 2.86 ^bc^	17.70 ± 0.86 ^a^	65.76 ± 2.15 ^b^	131.09 ± 2.00 ^c^
**RNAi-1**	16.28 ± 0.60 ^b^	1.27 ± 0.55 ^a^	43.54 ± 1.09 ^a^	31.38 ± 1.15 ^bc^	71.10 ± 1.05 ^a^	14.81 ± 0.59 ^b^	74.14 ± 4.38 ^a^	137.73 ± 2.39 ^b^
**RNAi-5**	16.92 ± 1.33 ^b^	1.60 ± 0.55 ^a^	42.83 ± 1.41 ^a^	32.77 ± 1.09 ^b^	58.47 ± 9.07 ^bc^	14.95 ± 0.86 ^b^	69.02 ± 5.69 ^ab^	137.68 ± 2.72 ^b^
**RNAi-7**	17.30 ± 1.17 ^b^	1.94 ± 2.75 ^a^	42.95 ± 0.69 ^a^	30.47 ± 1.54 ^bc^	66.73 ± 0.61 ^b^	13.46 ± 1.50 ^bc^	69.76 ± 3.85 ^ab^	141.16 ± 4.07 ^b^

At the flowering stage, the contents of crude protein (CP, %), ether extract (EE, %), neutral detergent fiber (NDF, %), acid detergent fiber (ADF, %), dry matter (DM, %), and crude ash (Ash, %) were measured and analyzed in RNAi, wild-type (WT), and overexpression (OE) plants, respectively, and the stem-to-leaf ratio (SLR, %) and relative feed value (RFV) were calculated. Different letters in the same column indicate statistically significant differences (*p* < 0.05), while identical letters or no letters indicate no significant difference (*p* > 0.05).

## Data Availability

The original contributions presented in this study are included in the Supplementary Materials. Further inquiries can be directed to the corresponding author.

## Supplementary Materials

The following supporting information can be downloaded at: https://www.mdpi.com/article/10.3390/plants15132028/s1. Table S1(1). Primer Sequences and Their Applications. Table S1(2). Primer Amplification System. Table S1(3). Primer PCR Protocol. Table S1(4). Reverse Transcription Reaction System. Table S2(1). T-Vector Ligation Reaction System. Table S2(2). Monoclonal Bacterial Culture PCR Reaction System. Table S2(3). Expression vector construction system. Table S2(4). Overexpression Vector Construction System. Table S2(5). RNA Interference (RNAi) Vector Construction System. Table S3. Phylogenetic mapping analysis of the AUX/IAA family. Table S4. Transcriptional Expression Levels of *MsARG4* in RNAi, Wild-Type (WT), and Overexpression (OE) at Different Developmental Stages. Table S5. Phenotypic Traits of RNAi, Wild-Type (WT), and Overexpression (OE) at the Branching, Budding and Flowering Stages. Table S6. Nutritional Quality Analysis of RNAi, Wild-Type (WT), and Overexpression (OE) at the Flowering Stage. Table S7(1). Content of Growth-Related Phytohormones in Leaf Tissues of Alfalfa. Table S7(2). Content of Growth-Related Phytohormones in Stem Tissues of Alfalfa. Table S8(1) Content of SS and SP in Stem Tissues of Alfalfa. Table S8(2). Content of SS and SP in Leaf Tissues of Alfalfa. Table S9. Protein sequences of genes used in the phylogenetic tree. Table S10. BLAST analysis of the MsARG4-RNAi target sequence in alfalfa ‘Zhongmu No.1’. Table S11(1). Morphometry of cellular structure in transverse section. Table S11(2). Morphometry of cellular structure in longitudinal section. Figure S1. Cell structures of stem tissues in RNAi, WT, and OE plants at different developmental stages.
