# Supplementary material for: The Auxin-Induced Protein Gene (MsARG4) Regulates Rapid Stem Elongation and Nutritional Quality Enhancement in Alfalfa
Source: Plants (Basel). 2026 Jun 30;15(13):2028. doi: 10.3390/plants15132028 (PMC13364422; doi:10.3390/plants15132028)
Supplement: Supplementary file 1 [file plants-15-02028-s001.zip › Figure S1.pdf]

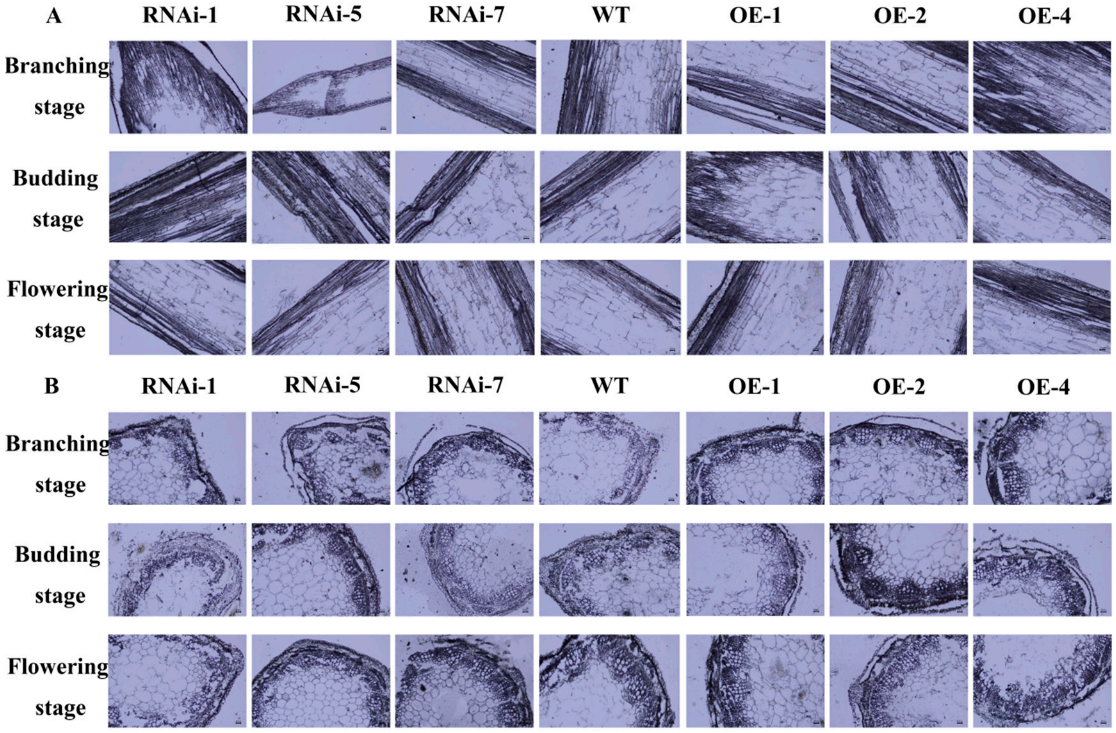

Figure S1. Cell structures of stem tissues in RNAi, WT, and OE plants at different developmental stages. The figures show the cell structure of stem tissues of different alfalfa lines at different growth stages. Figure A shows longitudinal sections of stem tissues from different plants at various growth stages; Figure B displays cross-sectional views of stem tissues from the different plants at different developmental stages. Scale: 50 μm.
