# Supplementary material for: The Auxin-Induced Protein Gene (MsARG4) Regulates Rapid Stem Elongation and Nutritional Quality Enhancement in Alfalfa
Source: Plants (Basel). 2026 Jun 30;15(13):2028. doi: 10.3390/plants15132028 (PMC13364422; doi:10.3390/plants15132028)
Supplement: Supplementary file 1 [file plants-15-02028-s001.zip › Table S1-8.pdf]

Table S1(1). Primer Sequences and Their Applications.

Primer ID	Primer Type	Primer Sequence	Purpose of the Sequence
MsARG4	F	GCACCAACCTATGAAGACAAG	Preliminary Gene Validation and Subsequent qRT-PCR Quantification
	R	CACCACAACCCAAACCTC	
MsARG4-OE	F	GAGCTGTACAAGTAATCTAGATGGAATTCAAGGCAACTGAGCTTAGATTGG	Overexpression Vector Construction
	R	GGACTCTAGGGACTAGTCCCTCATACACCACAACCCAAACCTCTAGC	
MsARG4-RNAi	F	CAGTGGTCTCAGATCTAAGGTTTCAAATGATGATCAACATGTGGAGAGTTC	Interference Vector Construction
	R	CGATGGTCTCACAGGTAGGTTGGTGCATATTCAGATCCCTTATAACC	
Sequencing primer	F	TAAGGTTTCAAATGATGATCAACATG	Sequencing of positive strains
	R	TAAGGTTTCAAATGATGATCAACATG	

Table S1(2). Primer Amplification System

Component	Usage Amount
cDNA Template	1μl
2×SanTaq PCR Mix	10μl
Forward and Reverse Primers	1μl
DEPC H_2O	8μl
Total Reaction System	20μl

Table S1(3). Primer PCR Protocol

Primer ID	Temperature	Time	Cycle Number
Primer1	94°C	5 min	1
	94°C	30 sec	35
	53°C	40 sec	35
	72°C	90 sec	35
	72°C	10 min	1
	4°C	10 min	1
Primer2/3	94°C	5 min	1
	94°C	30 sec	35
	50°C	45 sec	35
	72°C	34 sec	35
	72°C	10 min	1
	4°C	30 min	1

Table S1(4). Reverse Transcription Reaction System

Reaction Procedure	Component	Usage Amount
Genomic DNA Removal	5×gDNA Eraser Buffer	2μl
	gDNA Eraser	1μl
	Total RNA	1μg
	RNase Free dH_2O	6μl
Reverse Transcription	First-Step Reaction Mixture	10μl
	PrimeScript RT Enzyme Mix I	1μl
	5×PrimeScript Buffer 2	4μl
	RT Primer Mix	1μl

Reaction Procedure	Component	Usage Amount
	RNase Free dH_2O	4μl
	Total Reaction System	20μl

Table S2(1). T-Vector Ligation Reaction System

Component	Usage Amount
pMD19-T Vector	0.5μl
DNA Recovery Product	4.5μl
Solution I	5μl
Total Reaction System	10μl

Table S2(2). Monoclonal Bacterial Culture PCR Reaction System

Component	Usage Amount
cDNA	1μl
Forward and Reverse Primers	1μl
2 x SanTaq PCRMix	10μl
DEPC H_2O	8μl
Total Reaction System	20μl

Table S2(3). Expression vector construction system

Component	Usage Amount
Plasmid	5μl
10 × QuiWTCut Buffer	2μl
QuiWTCut™ BamH I	1μl
QuiWTCut™ Sma I	1μl
DEPC H_2O	11μl
Total Reaction System	20μl

Table S2(4). Overexpression Vector Construction System

Component	Usage Amount
OE-*MsARG4*	6μl
Buffer	1μl
PEG100-OE/PEG100-RNAi	1μl
T4 DNA Ligase	1.5μl
DEPC H_2O	0.5μl
Total Reaction System	10μl

Table S2(5). RNA Interference (RNAi) Vector Construction System

Component	Usage Amount
RNAi-*MsARG4*	6μl
Buffer	1μl
PEG100-OE/PEG100-RNAi	1μl
T4 DNA Ligase	1.5μl
DEPC H_2O	0.5μl
Total Reaction System	10μl

Table S3. Phylogenetic mapping analysis of the AUX/IAA family

Gene_ID	Species	Gene_Name
MsG0180004486.01	Medicago sativa L.	*MsIAA10*
MsG0480021676.01	Medicago sativa L.	*MsIAA20*
MsG0880047279.01	Medicago sativa L.	*MsIAA39*
MsG0180004158.01	Medicago sativa L.	*MsIAA6*
MsG0680035708.01	Medicago sativa L.	*MsIAA30*
MsG0480023832.01	Medicago sativa L.	*MsIAA24*
MsG0180003906.01	Medicago sativa L.	*MsIAA4*
MsG0480018452.01	Medicago sativa L.	*MsIAA18*
MsG0780040698.01	Medicago sativa L.	*MsIAA35*
MsG0180004270.01	Medicago sativa L.	*MsIAA8*
MsG0780040694.01	Medicago sativa L.	*MsIAA33*
MsG0280011282.01	Medicago sativa L.	*MsIAA16*
MsG0580028330.01	Medicago sativa L.	*MsIAA26*
MsG0180001660.01	Medicago sativa L.	*MsIAA2*
MsG0780040693.01	Medicago sativa L.	*MsIAA32*
MsG0180003907.01	Medicago sativa L.	*MsIAA5*
MsG0180004272.01	Medicago sativa L.	*MsIAA9*
MsG0180004160.01	Medicago sativa L.	*MsIAA7*
NP_001329482.1	*Arabidopsis_thaliana*	*NPIAA14*
NP_001329438.1	*Arabidopsis_thaliana*	*NPIAA29*
NP_567891.1	*Arabidopsis_thaliana*	*NPIAA29*
NP_001329484.1	*Arabidopsis_thaliana*	*NPIAA14*
NP_188945.1	*Arabidopsis_thaliana*	*NPIAA7*
NP_187124.1	*Arabidopsis_thaliana*	*NPIAA16*
NP_182222.1	*Arabidopsis_thaliana*	*NPIAA20*
NP_175692.1	*Arabidopsis_thaliana*	*NPIAA6*
NP_193191.2	*Arabidopsis_thaliana*	*NPIAA14*
NP_173011.1	*Arabidopsis_thaliana*	*NPIAA5*
NP_171921.1	*Arabidopsis_thaliana*	*NPIAA17*
NP_001329483.1	*Arabidopsis_thaliana*	*NPIAA14*
NP_188173.1	*Arabidopsis_thaliana*	*NPIAA19*
NP_191769.1	*Arabidopsis_thaliana*	*NPIAA30*
NP_001325417.1	*Arabidopsis_thaliana*	*NPIAA20*
NP_001327812.1	*Arabidopsis_thaliana*	*NPIAA30*
NP_851275.1	*Arabidopsis_thaliana*	*NPIAA9*
NP_001318885.1	*Arabidopsis_thaliana*	*NPIAA9*
NP_188943.1	*Arabidopsis_thaliana*	*NPIAA2*
NP_001326722.1	*Arabidopsis_thaliana*	*NPIAA2*
NP_193192.1	*Arabidopsis_thaliana*	*NPIAA1*
NP_171920.1	*Arabidopsis_thaliana*	*NPIAA3*
NP_001322162.1	*Arabidopsis_thaliana*	*NPIAA3*
NP_199183.1	*Arabidopsis_thaliana*	*NPIAA4*

Table S4. Transcriptional Expression Levels of *MsARG4* in RNAi, Wild-Type (WT), and Overexpression (OE) at Different Developmental Stages

strain<br>stage	Branching stage	Budding stage	Flowering stage
	0.147965561	0.420448208	0.475219739
RNAi-1	0.129707457	0.363493129	0.520029967
	0.127921736	0.346277367	0.527289315
	0.22298213	0.326842312	0.463294031
RNAi-5	0.19144475	0.360149215	0.535886731
	0.232451236	0.298678784	0.38958229
	0.194341601	0.355190935	0.876605721
RNAi-7	0.32911568	0.30495466	0.435275282
	0.197054509	0.352737452	0.281264621
	0.976031761	0.976031761	0.976031761
WT-1	0.996540263	0.996540263	0.996540263
	0.867538687	0.867538687	0.867538687
	1.113421618	1.113421618	1.113421618
WT-2	1.113421618	1.113421618	1.113421618
	0.955945318	0.955945318	0.955945318
	1.073012339	1.073012339	1.073012339
WT-3	1.095558609	1.095558609	1.095558609
	1.241140694	1.241140694	1.241140694
	15.24220797	61.25121965	127.7045986
OE-1	12.72858374	65.64743177	115.8943694
	15.77972327	60.40795601	94.79024335
	25.45716748	67.80563804	141.2068892
OE-2	23.91758798	60.26854511	154.5218095
	29.04061297	61.10986265	156.6788535
	29.27640831	85.42975067	170.2683724
OE-4	32.71015446	100.1949973	124.6437442
	30.30883862	81.94969454	122.927737

Table S5. Phenotypic Traits of RNAi, Wild-Type (WT), and Overexpression (OE) at the Branching, Buddingand Flowering Stages

stage	strain	PH	SD	BN	LBN	IN	IL
Branching stage	OE-1	20.8	2.2	25	6	2	5.76
		21.3	2.1	26	6	1	5.34
		22.1	2.1	27	4	2	4.97
	OE-2	18.5	2.1	27	5	1	4.49
		17.9	2.0	28	6	1	4.32
		19.2	2.1	27	5	2	5.1
	OE-4	25.3	2.2	28	5	3	4.98
		24.9	2.1	28	4	3	5.13
		27.3	2.1	31	6	2	4.56
	WT	15.1	1.7	20	3	3	3.45
		17.2	1.6	18	2	3	3.23
		15.3	1.6	21	4	4	3.54
	RNAi-1	14.7	1.7	17	3	4	2.66
		14.3	1.5	16	2	4	2.54
		15.1	1.5	17	2	3	3.01
	RNAi-5	14.3	1.4	17	2	3	3.26
		14.8	1.4	16	3	4	2.94
		16.2	1.3	18	2	4	2.89
	RNAi-7	18.2	1.6	17	3	3	3.05
		15.3	1.5	16	2	4	2.84
		14.1	1.5	14	2	4	2.56
Budding stage	OE-1	49.1	2.4	28	12	8	5.08
		50.3	2.3	27	12	8	5.46
		47.2	2.3	25	14	7	4.91
	OE-2	39.4	2.3	29	11	8	4.57
		38.5	2.3	28	13	7	4.32
		43.5	2.2	29	11	9	4.94
	OE-4	60.5	2.2	31	11	8	6.26
		57.3	2.2	28	11	8	6.32
		58.2	2.4	29	12	7	5.98
	WT	39.7	1.7	19	7	7	4.36
		40.5	1.5	20	10	9	4.32
		46.2	1.6	23	9	8	4.85
	RNAi-1	38.4	1.7	18	7	7	2.34
		37.4	1.5	17	7	7	2.79
		39.3	1.7	15	8	8	3.01
	RNAi-5	25.6	1.4	20	8	6	2.42
		28.4	1.5	22	8	6	2.54

		24.1	1.4	24	6	7	3.10
		31.5	1.6	16	7	6	2.97
	RNAi-7	28.7	1.4	15	9	8	2.46
		29.5	1.5	17	9	6	2.72
Flowering stage	OE-1	59.2	2.5	31	23	11	5.15
		60.1	2.5	30	20	10	5.64
		57.3	2.3	27	23	11	4.93
	OE-2	54.4	2.3	30	16	8	4.87
		49.3	2.2	27	18	10	5.02
		52.1	2.3	31	18	10	4.93
	OE-4	70.9	2.4	33	20	11	6.36
		69.1	2.4	31	18	10	6.21
		71.3	2.2	29	22	11	5.74
	WT	51.2	1.7	20	9	11	4.36
		52.5	1.6	23	7	10	4.23
		50.7	1.7	24	11	10	4.95
	RNAi-1	39.4	1.6	18	10	11	2.52
		40.9	2.1	17	5	13	3.14
		45.7	1.8	18	6	10	2.94
	RNAi-5	40.5	1.4	25	10	12	3.2
		46.2	1.8	25	8	11	3.49
		49.3	1.4	23	8	10	4.13
	RNAi-7	33.7	1.8	17	10	11	3.02
		39.5	1.3	16	9	12	4.12
		37.2	1.4	17	6	11	3.85

Table S6. Nutritional Quality Analysis of RNAi, Wild-Type (WT), and Overexpression (OE) at the Flowering Stage

strain Nutrition Index	ASH (%)	NDF (%)	ADF (%)	CP (%)	EE (%)	SLR (%)	DM (%)	RFV
OE-1	12.76	41.16	30.12	25.69	2.59	57.66	47.14	147.90
	11.07	40.25	29.13	23.69	4.52	53.10	46.48	153.03
	12.90	42.10	27.14	21.37	2.31	55.15	47.63	149.72
OE-2	10.75	40.27	27.63	21.42	4.20	56.60	39.56	155.65
	13.63	41.17	28.46	24.43	3.40	55.27	43.10	150.77
	11.58	39.99	27.16	23.75	2.07	55.37	40.51	157.59
OE-4	12.95	39.65	29.12	22.59	4.31	58.55	55.85	155.36
	14.12	40.41	28.14	21.59	3.43	57.22	53.65	154.20
	14.37	41.07	29.91	23.40	2.95	56.83	52.87	148.60
WT	17.58	42.58	37.59	18.93	1.92	68.18	57.88	130.24
	16.91	43.06	37.07	17.31	3.14	64.06	62.21	129.66
	18.62	41.24	38.23	19.70	2.81	65.03	56.80	133.37
RNAi-1	15.33	44.73	30.14	16.21	1.40	76.52	69.90	136.04
	14.93	42.58	31.59	16.91	1.75	69.08	71.84	140.47
	14.17	43.32	32.41	15.71	0.67	76.81	71.56	136.69
RNAi-5	15.92	41.64	33.53	17.41	1.88	71.59	48.72	140.23
	14.63	42.46	33.26	17.93	0.97	62.50	60.03	138.01
	14.30	44.39	31.53	15.41	1.96	72.96	66.65	134.81
RNAi-7	13.89	43.20	29.13	17.22	2.76	69.23	67.13	142.57
	11.79	42.17	30.12	16.17	2.42	66.20	67.03	144.34
	14.69	43.49	32.15	18.51	0.63	73.84	66.03	136.58

Table S7(1). Content of Growth-Related Phytohormones in Leaf Tissues of Alfalfa

stage	strain	IAA	CTK	GA	BR
Branching stage	OE-1	34.58542714	11.44836272	48.6961326	18.292044
		36.24371859	11.19647355	45.60220994	18.80701817
		35.36432161	11.80100756	46.87292818	17.36417033
	OE-2	35.94221106	10.16372796	55.60220994	17.30401265
		38.75628141	13.31234257	53.33701657	18.87240107
		39.76130653	11.90176322	54.60773481	21.19916741
	OE-4	33.58040201	11.2720403	34.33149171	18.10258508
		31.72110553	9.609571788	35.10497238	22.96067818
		32.52512563	11.17128463	30.4640884	19.81199202
	WT	32.09798995	8.979848866	28.30939227	22.3319684
		28.42964824	8.753148615	26.81767956	20.90691489
		28.60552764	8.652392947	29.41436464	22.92087036
	Ri-1	26.89698492	7.82115869	27.31491713	23.64808757
		24.08291457	8.324937028	27.14917127	22.44852573
		25.18844221	7.720403023	28.64088398	24.14567246
	Ri-5	24.71105528	8.62720403	27.14917127	23.16075672
		23.83165829	8.576826196	26.54143646	23.24127547
		24.25879397	8.55163728	25.38121547	22.60488237
	Ri-7	24.23366834	7.695214106	25.71270718	23.89558488
		22.55025126	7.267002519	29.13812155	23.56615944
		22.75125628	8.677581864	27.5359116	23.28163975
Budding stage	OE-1	43.75628141	8.753148615	34.11049724	25.48009904
		44.53517588	8.879093199	36.43093923	26.93497181
		44.93718593	8.727959698	36.48618785	31.43312207
	OE-2	45.2638191	10.39042821	40.1878453	25.5243516
		45.86683417	9.483627204	43.50276243	30.20331177
		46.82160804	9.005037783	38.4198895	29.78692777
	OE-4	46.59547739	9.231738035	30.74033149	27.6932695
		43.85678392	9.483627204	30.62983425	26.3799061
		46.19346734	9.282115869	32.45303867	23.85415617
	WT	39.9120603	8.22418136	25.93370166	23.64808757
		37.60050251	7.871536524	26.32044199	23.16075672
		36.99748744	7.770780856	27.59116022	22.80186052
	Ri-1	35.13819095	6.637279597	22.89502762	19.70912411
		35.41457286	6.435768262	23.39226519	15.8384575
		35.11306533	6.057934509	20.35359116	16.28435608
	Ri-5	35.96733668	5.579345088	22.28729282	15.62010793
		33.17839196	6.536523929	22.78453039	16.42625739
		34.81155779	6.889168766	22.95027624	15.89352015
	Ri-7	36.99748744	6.360201511	27.8121547	17.03587183
		36.84673367	7.619647355	27.92265193	16.77188608
		36.99748744	7.871536524	31.45856354	15.78358561

		13.75628141	18.8790932	34.71823204	22.2932506
	OE-1	13.58040201	18.47607053	35.60220994	21.72047397
		13.55527638	19.28211587	34.99447514	21.985915
		13.25376884	17.64483627	36.20994475	22.44852573
	OE-2	13.90703518	16.9395466	35.04972376	22.37075345
		13.65577889	20.13853904	35.65745856	23.12060202
		18.42964824	31.4231738	42.28729282	19.74335393
	OE-4	19.96231156	34.67254408	36.43093923	22.1390495
		20.13819095	36.58690176	38.19889503	20.05410992
		13.20351759	15.1511335	34.82872928	19.57279726
Flowering stage	WT	12.04773869	15.68010076	33.44751381	18.74186179
		11.6959799	15.4534005	31.23756906	18.90517771
		9.962311558	11.95214106	27.92265193	17.03587183
	Ri-1	11.14321608	12.53148615	25.71270718	17.33406539
		9.610552764	10.03778338	24.77348066	17.91508848
		9.610552764	9.911838791	25.38121547	17.12478722
	Ri-5	8.077889447	8.450881612	24.60773481	16.97685153
		11.01758794	8.073047859	24.33149171	17.09509731
		11.67085427	12.95969773	26.32044199	18.61222543
	Ri-7	12.77638191	10.89420655	28.80662983	16.34096891
		12.42462312	10.89420655	28.6961326	16.48336354

Table S7(2). Content of Growth-Related Phytohormones in Stem Tissues of Alfalfa

stage	strain	IAA	CTK	GA	BR
		37.12311558	10.39042821	32.89502762	30.73198641
	OE-1	38.78140704	10.18891688	33.17127072	28.17800869
		40.0879397	10.59193955	34.71823204	29.12251151
		37.27386935	11.09571788	33.00552486	24.39837741
	OE-2	37.09798995	10.21410579	30.85082873	24.44075129
		39.93718593	11.04534005	30.13259669	24.86855885
		37.09798995	10.79345088	35.98895028	24.9550148
	OE-4	36.79648241	11.09571788	33.66850829	26.98175112
		37.29899497	11.24685139	31.62430939	24.61098402
Branching stage		36.21859296	9.886649874	29.41436464	22.96067818
	WT	35.69095477	9.710327456	28.75138122	25.5243516
		36.54522613	8.476070529	28.86187845	23.04050135
		32.39949749	7.392947103	27.70165746	21.05253402
	Ri-1	32.39949749	8.526448363	25.82320442	20.44057293
		29.2839196	9.483627204	26.37569061	21.30981231
		32.09798995	7.141057935	25.54696133	23.32207413
	Ri-5	32.85175879	8.274559194	27.5359116	19.91539682
		34.78643216	6.738035264	26.37569061	21.38389621
	Ri-7	29.03266332	9.609571788	25.54696133	21.79598555

		27.39949749	8.879093199	26.81767956	21.30981231
		27.14824121	9.408060453	28.36464088	22.21601627
Budding stage	OE-1	41.14321608	11.67506297	31.84530387	25.83627897
		39.9120603	11.07052897	33.44751381	24.568315
		39.58542714	11.14609572	30.4640884	26.8882736
	OE-2	46.59547739	11.75062972	35.98895028	24.78240243
		44.58542714	13.79093199	36.43093923	25.92609921
		47.60050251	12.05289673	32.83977901	27.31148903
	OE-4	39.08291457	10.44080605	31.23756906	23.8127993
		39.30904523	9.861460957	34.71823204	23.36257874
		40.36432161	11.87657431	33.06077348	23.12060202
	WT	37.12311558	10.06297229	30.13259669	23.04050135
		37.77638191	9.785894207	29.85635359	22.84146165
		38.4798995	10.01259446	29.96685083	22.96067818
	Ri-1	31.5201005	7.896725441	26.59668508	20.08893889
		30.06281407	7.518891688	26.43093923	18.41945025
		32.47487437	8.879093199	26.59668508	16.83019381
	Ri-5	32.75125628	7.392947103	26.54143646	18.10258508
		32.09798995	7.115869018	26.76243094	17.54590111
		33.95728643	7.216624685	26.32044199	20.79836192
	Ri-7	36.34422111	8.350125945	28.25414365	19.88086873
		34.61055276	8.95465995	26.26519337	20.3344413
		37.5	9.307304786	27.75690608	23.20098117
Flowering stage	OE-1	11.39447236	7.921914358	28.53038674	18.26033038
		10.21356784	7.770780856	28.4198895	18.80701817
		10.48994975	8.123425693	27.86740331	19.50498786
	OE-2	10.99246231	8.173803526	25.60220994	18.5799567
		10.38944724	8.375314861	26.20994475	17.88402839
		9.912060302	5.856423174	25.7679558	18.51558696
	OE-4	11.99748744	10.44080605	27.48066298	17.82206968
		11.19346734	7.619647355	27.86740331	18.90517771
		11.54522613	8.828715365	28.97237569	19.53886314
	WT	8.781407035	6.788413098	24.49723757	17.45479921
		8.881909548	6.158690176	23.72375691	18.07119993
		8.404522613	4.924433249	24.8839779	17.66811039
	Ri-1	7.876884422	3.916876574	22.61878453	16.68480302
		7.726130653	3.38790932	21.23756906	16.59817212
		6.922110553	3.992443325	22.95027624	16.97685153
	Ri-5	7.173366834	3.010075567	24.33149171	16.34096891
		7.474874372	3.110831234	24.77348066	15.78358561
		6.922110553	3.236775819	23.94475138	15.78358561
	Ri-7	8.530150754	5.226700252	24.05524862	16.14368062
		8.278894472	4.345088161	23.72375691	15.21878319
		8.153266332	4.622166247	22.50828729	15.70163401

Table S8(1) Content of SS and SP in Stem Tissues of Alfalfa

stage	strain	SS	SP
Branching stage	OE-1	6.765261628	0.337469194
		7.114098837	0.382935328
		7.288517442	0.392708422
	OE-2	5.260901163	0.320472508
		5.369912791	0.303050905
		5.587936047	0.338319028
	OE-4	7.615552326	0.329395768
		7.833575581	0.338743945
		8.029796512	0.379960908
	WT	2.775436047	0.290728308
		2.928052326	0.289028639
		3.058866279	0.323871845
	Ri-1	1.096656977	0.243987422
		1.162063953	0.2235914
		1.641715116	0.181524603
	Ri-5	1.837936047	0.189173111
		2.16497093	0.131809297
		2.099563953	0.100365429
	Ri-7	1.401889535	0.290303391
		1.598110465	0.269907368
		1.77252907	0.263533611
Budding stage	OE-1	6.242005814	0.674853404
		5.871366279	0.671454066
		6.26380814	0.787881363
	OE-2	4.955668605	0.475992182
		4.97747093	0.673578652
		5.064680233	0.477266933
	OE-4	6.678052326	0.393983173
		6.503633721	0.468343673
		6.787063953	0.484490524
	WT	3.952761628	0.39440809
		3.909156977	0.409705108
		3.538517442	0.411404776
	Ri-1	3.800145349	0.224441234
		3.429505814	0.284354551
		3.255087209	0.269907368
	Ri-5	3.146075581	0.365938642
		3.080668605	0.342993116
		2.884447674	0.343842951

		3.473110465	0.357865216
	Ri-7	3.84375	0.377411405
		3.255087209	0.341293448
Flowering stage		9.951671512	0.468343673
	OE-1	10.34411337	0.501912127
		10.63844477	0.495963287
		12.40443314	0.679527492
	OE-2	12.73146802	0.676128155
		13.22202035	0.678252741
		11.16170058	0.655732132
	OE-4	10.63844477	0.708846775
		10.99818314	0.695674344
		9.493822674	0.388884168
	WT	8.741642442	0.419053285
		8.97056686	0.395682842
		8.054869186	0.354465879
	Ri-1	8.807049419	0.345967536
		9.101380814	0.341293448
		4.359375	0.266508031
	Ri-5	4.653706395	0.254610351
		4.686409884	0.289453557
		5.307776163	0.292852894
	Ri-7	5.405886628	0.272031954
		5.602107558	0.277130959

Table S8(2). Content of SS and SP in Leaf Tissues of Alfalfa

stage	strain	SS	SP
Branching stage		8.465843023	1.076400102
	OE-1	8.792877907	1.047930654
		9.07630814	1.109968556
		9.316133721	1.013937282
	OE-2	9.66497093	0.862241863
		9.425145349	0.979094077
		7.550145349	0.924279765
	OE-4	8.160610465	1.066202091
		7.724563953	1.104019716
		5.544331395	0.760686666
	WT	5.631540698	0.811676723
		5.32630814	0.815500977
		5.021075581	0.744964732
	Ri-1	5.064680233	0.801053795
		4.890261628	0.785756777

	Ri-5	4.432412791	0.795529872
		4.170784884	0.764086003
		4.38880814	0.79722954
	Ri-7	4.367005814	0.866915951
		4.519622093	0.810401972
		4.519622093	0.766210589
Budding stage	OE-1	8.705668605	1.050480156
		9.207122093	1.073425682
		8.858284884	1.09892071
	OE-2	9.752180233	1.319877624
		9.621366279	1.386164698
		10.18822674	1.190277896
	OE-4	8.596656977	1.503441829
		8.945494186	1.295657347
		8.596656977	1.417183649
	WT	7.528343023	0.995665845
		7.898982558	1.09892071
		7.550145349	0.990991757
	Ri-1	5.435319767	0.530381576
		5.675145349	0.561400527
		4.97747093	0.593269312
	Ri-5	6.198401163	0.906008328
		5.849563953	0.841420923
		5.784156977	0.906008328
	Ri-7	7.04869186	0.953599048
		6.721656977	0.994816011
		6.438226744	0.982918331
Flowering stage	OE-1	10.40952035	1.207699499
		9.657340116	1.207699499
		9.657340116	1.199626073
	OE-2	12.5025436	1.202175576
		13.87609012	1.174131044
		13.48364826	1.192402482
	OE-4	11.32521802	1.141837342
		10.80196221	1.183054304
		9.493822674	1.157134359
	WT	8.054869186	1.072150931
		7.531613372	1.099345628
		6.714026163	1.072150931
	Ri-1	5.700218023	1.055579162
		6.844840116	1.055154245
		5.994549419	1.065352256
	Ri-5	7.531613372	1.055154245
		7.008357558	1.012662531

	7.597020349	1.017336619
	7.269985465	1.022010708
Ri-7	7.597020349	1.061103085
	7.335392442	1.037732642
