# Supplementary material for: The Auxin-Induced Protein Gene (MsARG4) Regulates Rapid Stem Elongation and Nutritional Quality Enhancement in Alfalfa
Source: Plants (Basel). 2026 Jun 30;15(13):2028. doi: 10.3390/plants15132028 (PMC13364422; doi:10.3390/plants15132028)
Supplement: Supplementary file 1 [file plants-15-02028-s001.zip › Table S10.pdf]

Target sequence:

TAAGGTTTCAAATGATGATCAACATGTGGAGAGTTCTTCTGCTGCCCCTCCTGCCAAG
GCAAAGATAGTTGGGTGGCCACCAATTAGGTCTTACAGGAAAAACACTTTGCAAGAG
GCTGAAGTTGGTGGGATCTATGTGAAAGTGAGCATGGATGGAGCTCCTTACCTTAGA
AAGATTGACTTGAGGATCTATGGAGGCTATCCAGAACTTCTCAAAGCTCTAGAAACC
ATGTTCAAATTGACCATAGGTGAGTATTCTGAAAGAGAGGGTTATAAGGGATCTGAA
TATGCACCAACCTA

MsARG4 **sequence:**

ATGGAATTCAAGGCAACTGAGCTTAGATTGGGATTACCAGGGACAGATGAAAAGGAGA
TGAAAACAATACATGGTAGTGTTGTTAAGAATAACAAAAGACAATTACCTCAAACTTCT
GAAGAATCTGTTTCAATTTCTAAGGTTTCAAATGATGATCAACATGTGGAGAGTTCTTC
TGCTGCCCCTCCTGCCAAGGCAAAGATAGTTGGGTGGCCACCAATTAGGTCTTACAGG
AAAAACACTTTGCAAGAGGCTGAAGTTGGTGGGATCTATGTGAAAGTGAGCATGGATG
GAGCTCCTTACCTTAGAAAGATTGACTTGAGGATCTATGGAGGCTATCCAGAACTTCTC
AAAGCTCTAGAAACCATGTTCAAATTGACCATAGGTGAGTATTCTGAAAGAGAGGGTT
ATAAGGGATCTGAATATGCACCAACCTATGAAGACAAGGATGGTGACTGGATGCTAGTT
GGAGATGTTCCATGGGACATGTTTGTGACTTCCTGCAAAAGACTAAGAATCATGAAAG
GCACAGAAGCTAGAGGTTTGGGTTGTGGTGTATGA

The target sequences are all 300 bp in length and perfectly match the nucleotide positions 138 bp-437 of the *MsARG4* sequence.

Table S10. BLAST analysis of the *MsARG4*-RNAi target sequence in alfalfa 'Zhongmu No.1'

Number	contig	query	family	length	e-value	score	ident %
1	MsG0280011282.01.T01	seq1	AUX/IAA	1002	1.8544E-04	51.0	76.2
2	MsG0580028330.01.T01	seq1	AUX/IAA	1107	1.8544E-04	51.0	79.2

A BLAST alignment was performed between the target sequence and the 'Zhongmu No.1' genome (http://alfalfagedb.liu-lab.com/genome_zm01/) with an E-value threshold of 0.001. The results, presented in Table S10, show that the target sequence exhibits high specificity within the genome, suggesting that the RNAi vector constructed based on this sequence possesses strong target specificity and is suitable for further experiments.
