# Supplementary material for: The Auxin-Induced Protein Gene (MsARG4) Regulates Rapid Stem Elongation and Nutritional Quality Enhancement in Alfalfa
Source: Plants (Basel). 2026 Jun 30;15(13):2028. doi: 10.3390/plants15132028 (PMC13364422; doi:10.3390/plants15132028)
Supplement: Supplementary file 1 [file plants-15-02028-s001.zip › Table S11.pdf]

Table S11(1). Morphometry of cellular structure in transverse section

Stage	line	Area(μm^2)	Perimeter(μm)	Feret' s diameter(μm)
Branching stage	OE-1	448.15^{Ba}	75.58^{Bb}	23.88^{Cb}
	OE-2	481.18^{Aa}	95.05^{Aa}	31.37^{Aa}
	OE-4	507.01^{Aa}	91.58^{ABa}	30.44^{Aa}
	WT	239.11^{Ab}	62.80^{Ac}	19.97^{Ac}
	RNAi-1	198.18^{Ab}	64.00^{Abc}	20.32^{ABc}
	RNAi-5	211.09^{Ab}	60.21^{Bc}	20.18^{Ac}
	RNAi-7	227.96^{Ab}	60.56^{Ac}	20.28^{ABc}
Budding stage	OE-1	486.79^{Ba}	90.11^{ABa}	28.92^{Ba}
	OE-2	542.51^{Aa}	96.55^{Aa}	31.67^{Aa}
	OE-4	361.03^{Bb}	70.42^{Bb}	22.66^{Bb}
	WT	236.85^{Ac}	62.44^{Abc}	20.28^{Abc}
	RNAi-1	193.63^{Ac}	53.48^{Bc}	18.38^{Bc}
	RNAi-5	205.50^{Ac}	69.32^{Ab}	21.76^{Ab}
	RNAi-7	219.34^{Ac}	57.45^{Ac}	18.16^{Bc}
Flowering stage	OE-1	648.37^{Aab}	103.01^{Aab}	33.57^{Aa}
	OE-2	438.85^{Abc}	92.39^{Ab}	28.88^{Aa}
	OE-4	772.16^{Aa}	116.78^{Aa}	32.74^{Aa}
	WT	221.85^{Ac}	65.34^{Ac}	21.73^{Ab}
	RNAi-1	224.53^{Ac}	62.52^{Ac}	21.31^{Ab}
	RNAi-5	242.21^{Ac}	61.91^{Bc}	21.51^{Ab}
	RNAi-7	235.10^{Ac}	63.66^{Ac}	21.57^{Ab}

Different lowercase letters indicate significant differences among lines at the same stage ($P < 0.05$); different uppercase letters indicate significant differences within the same line at different stages ($P < 0.05$); and identical letters denote no significant difference ($P > 0.05$).

Table S11(2). Morphometry of cellular structure in longitudinal section

Stage	line	Area(μm^2)	Perimeter(μm)	Feret' s diameter(μm)
Branching stage	OE-1	425.76^{Ba}	123.01^{Ba}	35.93^{Abc}
	OE-2	441.03^{Ba}	106.07^{Aa}	30.63^{Bc}
	OE-4	764.42^{Ba}	163.86^{Aa}	46.33^{Aab}
	WT	394.03^{Aa}	125.70^{Aa}	46.53^{Aab}
	RNAi-1	351.60^{Aa}	93.73^{Aa}	25.24^{Ac}
	RNAi-5	208.18^{Ba}	90.22^{Aa}	29.82^{Bc}
	RNAi-7	439.55^{Aa}	149.77^{Aa}	51.65^{Aa}
Budding stage	OE-1	649.74^{Bb}	155.36^{Aa}	50.63^{Aa}
	OE-2	1292.42^{Aa}	185.95^{Aa}	54.51^{Aa}
	OE-4	1230.51^{Aa}	146.92^{Aa}	41.78^{Aa}
	WT	568.81^{Ab}	134.26^{Aa}	40.63^{Aa}
	RNAi-1	500.01^{Ab}	150.62^{Aa}	47.79^{Aa}
	RNAi-5	461.61^{ABb}	129.49^{Aa}	43.24^{ABa}
	RNAi-7	664.95^{Ab}	130.08^{Aa}	35.73^{ABa}
Flowering stage	OE-1	1869.24^{Aa}	230.31^{Aa}	51.46^{Aa}
	OE-2	1415.10^{Aa}	146.92^{Aab}	51.81^{Aa}
	OE-4	1667.26^{Aa}	145.84^{Aab}	44.44^{Aa}
	WT	1305.21^{Aa}	130.50^{Aab}	38.02^{Aa}
	RNAi-1	634.19^{Ab}	134.31^{Aab}	47.80^{Aa}
	RNAi-5	491.89^{Ab}	138.31^{Aab}	50.18^{Aa}
	RNAi-7	833.95^{Ab}	96.56^{Ab}	31.81^{Ba}

Different lowercase letters indicate significant differences among lines at the same stage ($P < 0.05$); different uppercase letters indicate significant differences within the same line at different stages ($P < 0.05$); and identical letters denote no significant difference ($P > 0.05$).
